# Surface Magnetic Field and Phase Current Sensing of Steel Tape-Wrapped 3-Core MV Cables

**DOI:** 10.3390/s25082422

**Published:** 2025-04-11

**Authors:** Zuhan Zhao, Jixiong Xiao, Hang Li, Hang Wang

**Affiliations:** 1 Hubei Engineering Research Center for Safety Monitoring of New Energy and Power Grid Equipment, Hubei University of Technology, Wuhan 430068, China; zzh1292521736@163.com (Z.Z.);; 2 Wuhan Power Supply Company, State Grid Hubei Electric Power Co., Ltd., Wuhan 430000, China

**Keywords:** medium-voltage three-core cable, double-layer steel tape-wrapped structure, finite element simulation

## Abstract

The surface magnetic field of three-core cables is essential for estimating phase currents and locating the single-phase grounding faults. However, the double-layer steel tape-wrapped structure will shield the magnetic field, affecting the measurement of the surface magnetic field of the three-core cable. Disregarding the shielding effect of the steel tape during the measurement of the surface magnetic field of the cable leads to an erroneous phase current obtained from the inversion of the observed magnetic field. To measure the surface magnetic field and invert it to obtain three-phase currents accurately, a model to evaluate the shielding effect of the steel tape is proposed, and a differential evolutionary algorithm method is proposed to invert it to obtain the three-phase currents. The results indicate that the difference between the analytical and numerical solutions is below 5%. As the thickness of steel tape increases, the shielding coefficient also increases. The differential evolution algorithm can accurately estimate the three-phase currents. The differential evolutionary algorithm measured the current amplitude of phases A, B, and C to 354.62 A, 354.33 A, and 323.46 A, respectively. Additionally, the algorithm measured the current phase magnitudes to be −8.21°, −128.01°, and 111.87°. The maximum value of the amplitude error of the three-phase current is 4.83%, and the maximum value of the phase error is 8.01°.

## 1. Introduction

With the increasing urbanization of the country, the rate of cabling for the grid is constantly increasing [1,2]. The deep burial of urban cable lines makes cable fault localization extremely challenging. Failure to eliminate faults in time can easily cause multiple faults and even trigger large-scale power outages, which poses a severe threat to grid security. To ensure the power supply reliability of the power system, it is necessary to implement online monitoring of power cable operation. A more reliable method for directly monitoring the operation of three-core power cables is to measure the current of each cable core during actual operation. However, since the sum of the three-phase currents of a three-core cable is zero during stable operation, the total magnetic flux through any cross-section of the cable surface is also zero. The conventional induction method makes it challenging to measure phase currents. However, when current flows through a cable, a distribution of magnetic fields is generated on the cable’s surface. It is thus possible to obtain the cable core current by measuring the distribution of magnetic fields on the surface of the cable.

With the advancement of magnetic field sensor technology, there has been a growing number of reports on using sensors for current measurement in recent years [3]. In reference [4], researchers proposed a new magnetic field sensing technique to detect underground power cables and verified the method on simulation models of 11 kV and 132 kV underground power cables. Reference [5] presents a novel coreless current sensor unit for measuring the electrical current of circular conductors. The sensors do not need to be fixed to a conductor, which makes them more flexible. Reference [6] introduces a novel technique for conducting power current measurements. This technique has no iron core and consists of four Hall sensors, providing higher accuracy than conventional current transformers. Reference [7] proposes a theoretical and experimental evaluation method of crosstalk interference in a circular array of fluxgate sensors for current measurement. Reference [8] describes a coreless current transformer that utilizes the Hall effect and has multiple functions (a multifunctional HCT). The electronic circuit distinguishes between load and fault currents to perform metering and protective duties. By eliminating offset voltages and employing low-pass filtering techniques, the HCT achieves high accuracy (0.5 class) within IEC standard 60044-8 [9] for power system measurement and protection, thus overcoming core and saturation issues prevalent in traditional transformers. In reference [10], the influence of the inner displacement of the conductor on the accuracy of the circular array of magnetic field sensors was theoretically and experimentally shown. Reference [11] investigates a differential array of magnetic field sensors. In this differential approach, the Moore–Penrose inversion method (least squares inversion method) is used to obtain the set of currents at different positions inside the sensor by measuring the radial difference of the magnetic fields. Reference [12] presents a ring array of six small fluxgate magnetic field sensors to reliably detect current in flat conductors with great energy efficiency and bandwidth. The study obtained a current error of less than 1.5% by examining the effect of a flat conductor shape on measurement accuracy, advancing DC measurement techniques for microgrid applications. Reference [13] proposes a power current microsensor based on a Hall IC without an iron core. Reference [14] presents a contactless sensor design that enhances measurement precision and cost-effectiveness in distributed smart grid assessments. Reference [15] provides an in-depth analysis of the measurement errors of circular magnetic sensor arrays used for current measurements. By introducing a theoretical analysis based on vector mathematics, the paper explores the effect of conductor position variations (e.g., non-centering and non-perpendicularity) on measurement accuracy. Reference [16] presents an algorithm that can calculate the intensity of a DC flowing in a rectangular bus bar in the presence of crosstalk fields. Reference [17] presents a new method for accurately measuring AC currents in multi-core cables through the use of magnetic sensor arrays and differential evolutionary algorithms. Reference [18] proposes a method to measure the surface magnetic field of a three-core cable based on a ring Hall sensor. It measured the distribution of the surface magnetic field in the case of cable eccentricity.

Many scholars have studied three-core cables’ surface magnetic field measurements. These studies mainly focus on application and theory. Reference [19] proposed a numerical calculation method for the surface magnetic field of three-core cables with oil-filled tubes. This method considered the magnetic hysteresis characteristics of the steel tube and obtained the zero-sequence impedance of the three-core cable. In reference [20], the effects of skin and proximity were considered. The finite element method calculated the magnetic field distribution in a three-core submarine cable under industrial frequency current. In reference [21], Slovak scholars investigated the magnetic field distribution characteristics within the superconducting cable steel tape. The winding method of superconducting cable steel tape is similar to that of XLPE three-core cable steel tape. In references [22], an industrial frequency current measurement system experiment platform was constructed. References [23,24] propose an analytical method for magnetic field measurement of three-core cables, and an equation for the magnetic flux density at the intersection of the three cable centers with their respective phase centers has been given. However, the method does not consider the effects of skin and proximity. In the literature [25], three specific TSTC cables with the same strand spacing were designed, and the magnetic field’s distribution characteristics in the steel tape’s inner lining were investigated according to the T-A formula model and the H homogeneous formula.

Numerous scholars have conducted extensive studies on measuring the magnetic field of three-core cables. However, there are fewer studies on the magnetic field distribution on the surface of three-core cables under the double-layer steel tape-wrapped structure. Neglecting the shielding effect of the steel tape during the measurement of the surface magnetic field of the cable leads to an erroneous phase current obtained from the inversion of the observed magnetic field. In the references [26,27], a double-layer steel tape-wrapped structure in superconductivity is modeled and analyzed. However, only the magnetization loss in this structure was considered, and the shielding effect of the steel tape on the magnetic field generated by the conductor was not taken into account. The advancement of the shielding coefficient theory is relevant for measuring the magnetic field at the cable’s surface and for defending the external electromagnetic noise from the environment or criminals. Reference [28] discusses the significant issue of electromagnetic compatibility (EMC) in power electronics systems, with a specific emphasis on unmanned aerial vehicles (UAVs). Reference [29] provides a thorough analysis of intentional electromagnetic interference (IEMI) in power electronics, addressing test standards, conducted and radiated assault techniques, and protective measures. This document emphasizes Industry 4.0 and energy systems, outlining present issues and future research requirements for safeguarding critical infrastructure from emerging IEMI threats.

The proposed magnetic field sensing device offers low cost, low power consumption, and high sensitivity, while leveraging its non-invasive measurement capability and adaptability to harsh environments, presenting significant potential for diverse industrial applications. This encompasses the measurement of magnetic fields in high-voltage and superconducting cables, integration into power grid monitoring systems for real-time current detection, embedding within industrial machinery for predictive maintenance through electromagnetic signature analysis of motors and transformers, and deployment in smart manufacturing lines for accurate positioning of automated guided vehicles (AGVs) via magnetic marker tracking. In this paper, firstly, the magnetic shielding coefficients of the steel tape shield at different positions of the three-core cable under the double-layer steel tape-wrapped structure are deduced, and the system of equations for the relationship between magnetic induction intensity and current at the measuring point under the steel tape shield is constructed. Secondly, the three-dimensional three-core cable geometry model under the double-layer steel tape-wrapped structure was constructed in finite element simulation software. Then, a numerical calculation method for surface magnetic field inversion phase current based on the differential evolution algorithm is proposed. Finally, the experiment platform was built to measure the magnetic induction intensity on the cable surface using a ring Hall sensing device and compared it with the simulation results.

## 2. Modeling of Magnetic Field Sensing on the Surface of a Three-Core Cable Under a Double-Layer Steel Tape-Wrapped Structure

### 2.1. Shielding Coefficients and Magnetic Field Sensing Model on the Cable Surface

The shielding coefficient of the steel tape is initially derived in this section. Next, the magnetic induction intensity at the measurement location is determined while being shielded by the double-layer steel tape. Lastly, the shielding coefficients obtained from the analytical and simulation solutions are compared.

The steel tape can shield the magnetic field generated by the conductor because the magnetic permeability *μ* of the steel tape is much larger than the magnetic permeability *μ*_0_ of the air, so the magnetic resistance of the steel tape is minimal. When the steel tape is placed in the magnetic field generated by the conductor, the magnetic flux will mainly pass through the steel tape. In contrast, the magnetic flux through the air will be significantly reduced, thus playing a magnetic field shielding effect.

**Figure 1:**
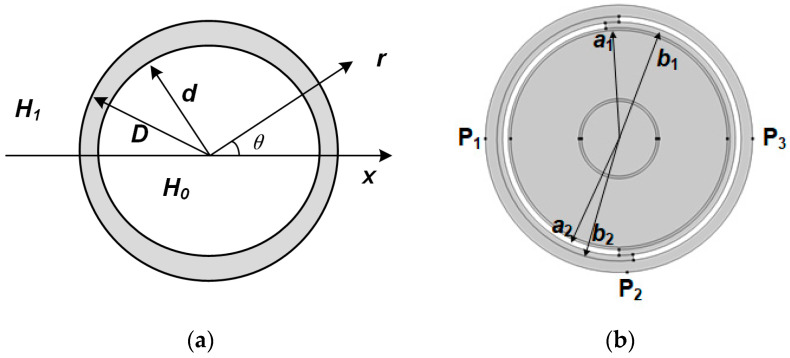
Single-layer shielding and double-layer wrap-around shielding (**a**) Single-layer shielding; (**b**) Double-layer shielding.

Figure 1a shows a single-layer shielding model, where the inner radius of the shielding layer of this geometrical model is size *d*, and the outer radius is size *D*. The relative permeability of the shielding layer is *μ*, assuming that the internal magnetic field is *H*_0_ and the external magnetic field is *H*_1_ after shielding. And *φ*_1_, *φ*_2_, and *φ*_3_ denote the magnetic potentials of the innermost, intermediate shielding, and the outermost layers, respectively. Then, the magnetic potentials of these three layers can be obtained by solving the Laplace equation [30].

(1) $$\nabla^{2}\varphi_{i}=0$$

Solving for the shielding coefficient of the transverse magnetic field of the shielding layer without considering the shielding coefficient of the axial magnetic field, we obtain:

(2) $$\frac{1}{r}\frac{\partial }{\partial r}(r\frac{\partial \varphi }{\partial r})+\frac{1}{r^{2}}\frac{\partial^{2}\varphi }{\partial \theta^{2}}=0$$

Applying the method of separated variables to the above equations, *φ*(*r*, *θ*) is viewed as the product of *φ*(*r*) and *φ*(*θ*), and so the partial differential Equation (2) is reduced to the solution of two ordinary differential equations:

(3) $$\frac{r}{\varphi }\frac{d^{2}\varphi }{dr^{2}}+\frac{r}{\varphi }\frac{d\varphi }{dr}=\lambda$$

(4) $$-\frac{1}{\varphi }\frac{d^{2}\varphi }{d\theta^{2}}=\lambda$$

where *λ* is a positive constant, the final general solution of Equation (2) is obtained as:

(5) $$\begin{array}{c} \varphi_{m}(r,\theta )=C_{1}r+D_{1}\\ +\sum_{n=1}^{\infty }r^{n}\left[A_{n}cos(n\theta )+B_{n}sin(n\theta )\right]\\ +\sum_{n=1}^{\infty }r^{-n}\left[C_{n}cos(n\theta )+D_{n}sin(n\theta )\right] \end{array}$$

where *A_n_*, *B_n_*, *C_n_*, *D_n_*, *C*_1_, and *D*_1_ are constants to be determined.

The boundary conditions are as follows:

(6) $$\begin{array}{c} r=d,\varphi_{1}=\varphi_{2},\frac{\partial \varphi_{1}}{\partial r}=\mu_{r}\frac{\partial \varphi_{2}}{\partial r}\\ r=D,\varphi_{2}=\varphi_{3},\mu_{r}\frac{\partial \varphi_{2}}{\partial r}=\frac{\partial \varphi_{3}}{\partial r}\\ r\to 0,\varphi_{3}=-H_{0}rcos\theta \end{array}$$

The shielding coefficients for a single layer of steel tape based on the boundary conditions above are as follows:

(7) $$ST=\frac{B_{0}}{B_{1}}=\frac{H_{0}}{H_{1}}=\frac{D^{2}{\left(\mu_{r}+1\right)}^{2}-d^{2}{\left(\mu_{r}-1\right)}^{2}}{4\mu_{r}D^{2}}$$

where *u_r_* is the relative magnetic permeability of the steel tape, and *ST* is the shielding coefficient.

The shielding coefficient formula of a single-layer steel tape structure can be used to derive the steel-wrapped shielding coefficient of a double-layer steel tape-wrapped structure.

As depicted in Figure 1b, the shielding coefficient of a cable under a double-layer steel tape-wrapped structure can be divided into three parts: (1) only the shielding of the inner layer of steel tape is considered; (2) only the shielding under the outer layer of steel tape is considered; (3) the shielding under the shielding of the inner and outer layers of steel tape overlay is considered.

(1) Only the shielding of the inner layer of steel tape is considered:

(8) $$ST_{1}=\frac{b_{1}^{2}{\left(\mu_{r}+1\right)}^{2}-a_{1}^{2}{\left(\mu_{r}-1\right)}^{2}}{4\mu_{r}b_{1}^{2}}$$

(2) Only the shielding under the outer layer of steel tape is considered:

(9) $$ST_{2}=\frac{b_{2}^{2}{\left(\mu_{r}+1\right)}^{2}-a_{2}^{2}{\left(\mu_{r}-1\right)}^{2}}{4\mu_{r}b_{2}^{2}}$$

(3) The shielding under the shielding of the inner and outer layers of steel tape overlay is considered, and therefore, the shielding coefficient is:

(10) $$ST_{3}=\frac{b_{2}^{2}{\left(\mu_{r}+1\right)}^{2}-a_{1}^{2}{\left(\mu_{r}-1\right)}^{2}}{4\mu_{r}b_{2}^{2}}$$

where *a*_1_, *b*_1_, *a*_2_, and *b*_2_ are the inner and outer radii of the inner and outer double-layer steel tape, respectively.

**Figure 2:**
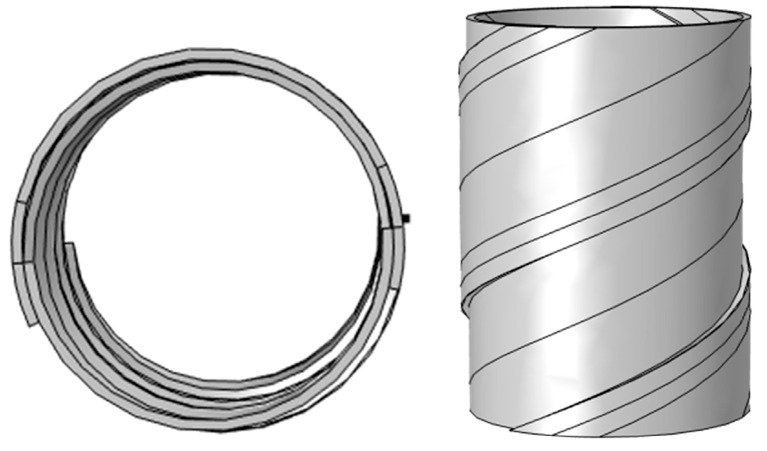
Three-dimensional modeling of the solenoid sheath.

The metal sheath of the cable can be regarded as solenoids stacked on top of each other, as shown in Figure 2.

The parametric equation in its cylindrical coordinates is:

(11) $$\left\{\begin{array}{c} x=\rho cos\theta \\ y=\rho sin\theta \\ z=z \end{array}\right.$$

where the column radius is *p* ∈ [*r* − 0.5*t*, *r* + 0.5*t*]. *t* is the thickness of the metal sheath. According to the Biot–Saval law, the core current *I* produces a magnetic induction intensity *B* at a distance *p* from the wire:

(12) $$B=\frac{\mu_{0}}{4\pi }\int_{\theta_{1}}^{\theta_{2}}\frac{Isin\theta d\theta }{p}=\frac{\mu_{0}I}{2\pi \rho }$$

**Figure 3:**
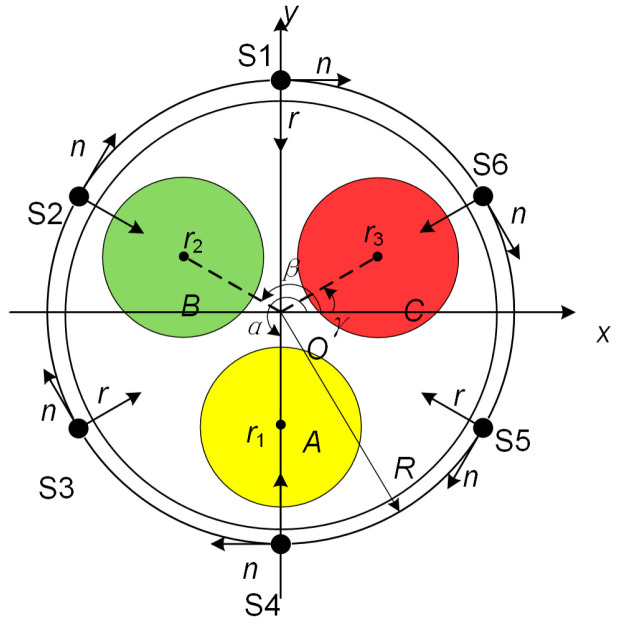
Six points from S1 to S6.

This article designed 6 Hall sensing arrays with a mutual difference of 60°, numbered S1–S6, and placed them on the surface of the three-core cable, as depicted in Figure 3. Point O is the center of the three-core cable. Points A, B, and C are the centers of phase A, B, and C cores. The distances from point O to the centers of each core are *r*_1_, *r*_2_, and *r*_3_, respectively. The radius of the cable is *R*. The angles between OA, OB, OC, and the positive direction of the *x*-axis are *α*, *β*, and *γ* respectively, with a mutual difference of 120°. From the trigonometric relationship, the coordinates of points A, B, and C can be obtained as (*r*_1_cos*α*, *r*_1_sin*α*), (*r*_2_cos*β*, *r*_2_sin*β*), (*r*_3_cos*γ*, *r*_3_sin*γ*).

According to Equation (12), the magnetic induction produced by the phase A current at the measuring location S1 is:

(13) $$B_{A}=\frac{\mu_{0}I_{A}}{2\pi \sqrt{R^{2}+r_{1}^{2}-2Rr_{1}\sin\alpha }}$$

The magnetic induction in the *n* direction is:

(14) $$B_{A-n}=\frac{\mu_{0}I_{A}\left(R-r_{l}sin\alpha \right)}{2\pi \left(R^{2}+r_{l}^{2}-2Rr_{l}\sin\alpha \right)}$$

The magnetic induction produced by the phase B current at the measuring location S1 is:

(15) $$B_{B}=\frac{\mu_{0}I_{B}}{2\pi \sqrt{R^{2}+r_{2}^{2}-2Rr_{2}\sin\left(\alpha -120{}^{\circ}\right)}}$$

The magnetic induction in the *n* direction is:

(16) $$B_{B-n}=\frac{\mu_{0}I_{B}\left(R-r_{2}sin(\alpha -120{}^{\circ})\right)}{2\pi \left(R^{2}+r_{2}^{2}-2Rr_{2}\sin\left(\alpha -120{}^{\circ}\right)\right)}$$

The magnetic induction produced by the phase C current at the measuring location S1 is:

(17) $$B_{C}=\frac{\mu_{0}I_{C}}{2\pi \sqrt{R^{2}+r_{3}^{2}-2Rr_{3}\sin\alpha (\alpha +120{}^{\circ})}}$$

The magnetic induction in the *n* direction is:

(18) $$B_{C-n}=\frac{\mu_{0}I_{C}\left(R-r_{3}sin(\alpha +120{}^{\circ})\right)}{2\pi \sqrt{R^{2}+r_{3}^{2}-2Rr_{3}\sin\alpha (\alpha +120{}^{\circ})}}$$

Based on the principle of superposition, the magnetic induction intensity at measurement site S1, along the tangential direction of the cable circumference, can be expressed as *B*_1_ = *B*_A-*n*_ + *B*_B-*n*_ + *B*_C-*n*_.

(19) $$B_{1}=\frac{\mu_{0}}{2\pi }(\frac{R-r_{1}sin\alpha }{R^{2}+{r_{1}}^{2}-2Rr_{1}sin\alpha }I_{A}+\frac{R-r_{2}sin(\alpha -120{}^{\circ})}{R^{2}+{r_{2}}^{2}-2Rr_{2}sin(\alpha -120{}^{\circ})}I_{B}+\frac{R-r_{3}sin(\alpha +120{}^{\circ})}{R^{2}+{r_{3}}^{2}-2Rr_{3}sin(\alpha +120{}^{\circ})}I_{C})$$

According to the principle of electromagnetic field, when three cores are connected to three-phase alternating current without steel tape shielding, the magnetic induction intensity *B* at the measurement points S1 to S6 is as follows:

(20) $$B_{2}=\frac{\mu_{0}}{2\pi }[\frac{R-r_{1}sin(\alpha -60{}^{\circ})}{R^{2}+{r_{1}}^{2}-2Rr_{1}sin(\alpha -60{}^{\circ})}I_{A}+\frac{R-r_{2}sin(\alpha -180{}^{\circ})}{R^{2}+{r_{2}}^{2}-2Rr_{2}sin(\alpha -180{}^{\circ})}I_{B}+\frac{R-r_{3}sin(\alpha +60{}^{\circ})}{R^{2}+{r_{3}}^{2}-2Rr_{3}sin(\alpha +60{}^{\circ})}I_{C}]$$

(21) $$B_{3}=\frac{\mu_{0}}{2\pi }[\frac{R-r_{1}sin(\alpha -120{}^{\circ})}{R^{2}+{r_{1}}^{2}-2Rr_{1}sin(\alpha -120{}^{\circ})}I_{A}+\frac{R-r_{2}sin(\alpha +120{}^{\circ})}{R^{2}+{r_{2}}^{2}-2Rr_{2}sin(\alpha +120{}^{\circ})}I_{B}+\frac{R-r_{3}sin\alpha }{R^{2}+{r_{3}}^{2}-2Rr_{3}sin\alpha }I_{C}]$$

(22) $$B_{4}=\frac{\mu_{0}}{2\pi }[\frac{R-r_{1}sin(\alpha -180{}^{\circ})}{R^{2}+{r_{1}}^{2}-2Rr_{1}sin(\alpha -180{}^{\circ})}I_{A}+\frac{R-r_{2}sin(\alpha +60{}^{\circ})}{R^{2}+{r_{2}}^{2}-2Rr_{2}sin(\alpha +60{}^{\circ})}I_{B}+\frac{R-r_{3}sin(\alpha -60{}^{\circ})}{R^{2}+{r_{3}}^{2}-2Rr_{3}sin(\alpha -60{}^{\circ})}I_{C}]$$

(23) $$B_{5}=\frac{\mu_{0}}{2\pi }[\frac{R-r_{1}sin(\alpha +120{}^{\circ})}{R^{2}+{r_{1}}^{2}-2Rr_{1}sin(\alpha +120{}^{\circ})}I_{A}+\frac{R-r_{2}sin\alpha }{R^{2}+{r_{2}}^{2}-2Rr_{2}sin\alpha }I_{B}+\frac{R-r_{3}sin(\alpha -120{}^{\circ})}{R^{2}+{r_{3}}^{2}-2Rr_{3}sin(\alpha -120{}^{\circ})}I_{C}]$$

(24) $$B_{6}=\frac{\mu_{0}}{2\pi }[\frac{R-r_{1}sin(\alpha +60{}^{\circ})}{R^{2}+{r_{1}}^{2}-2Rr_{1}sin(\alpha +60{}^{\circ})}I_{A}+\frac{R-r_{2}sin(\alpha -60{}^{\circ})}{R^{2}+{r_{2}}^{2}-2Rr_{2}sin(\alpha -60{}^{\circ})}I_{B}+\frac{R-r_{3}sin(\alpha +180{}^{\circ})}{R^{2}+{r_{3}}^{2}-2Rr_{3}sin(\alpha +180{}^{\circ})}I_{C}]$$

Considering the shielding coefficient, the magnetic induction intensity *B’* = *B*/*S*_T_ at the measurement points S1–S6 satisfies Equations (8)–(10).

(1) Only the shielding of the inner layer of steel tape is considered:

(25) $$B_{a}\prime =\frac{4\mu_{r}b_{1}^{2}}{b_{1}^{2}{\left(\mu_{r}+1\right)}^{2}-a_{1}^{2}{\left(\mu_{r}-1\right)}^{2}}B$$

(2) Only the shielding under the outer layer of steel tape is considered:

(26) $$B_{b}\prime =\frac{4\mu_{r}b_{2}^{2}}{b_{2}^{2}{\left(\mu_{r}+1\right)}^{2}-a_{2}^{2}{\left(\mu_{r}-1\right)}^{2}}B$$

(3) The shielding under the shielding of the inner and outer layers of steel tape overlay is considered, and therefore, the shielding coefficient is:

(27) $$B_{c}\prime =\frac{4\mu_{r}b_{2}^{2}}{b_{2}^{2}{\left(\mu_{r}+1\right)}^{2}-a_{1}^{2}{\left(\mu_{r}-1\right)}^{2}}B$$

### 2.2. Comparison of Analytical and Numerical Solutions for Shielding Coefficients

Construct a finite element simulation model as shown in Figure 1b, apply a power frequency current of 100 A to the wire core, and set the magnetic permeability of the steel tape to 1, 200, 400, 600, and 800, respectively. Then, numerical methods are used to calculate the magnetic induction intensity at points P_1_ (shielded only by the outer steel tape), P_2_ (shielded by the inner and outer double-layered steel tape), and P_3_ (shielded only by the outer steel tape). The results are shown in Table 1.

Table 1 shows the magnitude of the magnetic induction intensity at P_1_, P_2_, and P_3_ under different magnetic permeabilities.

**Figure 4:**
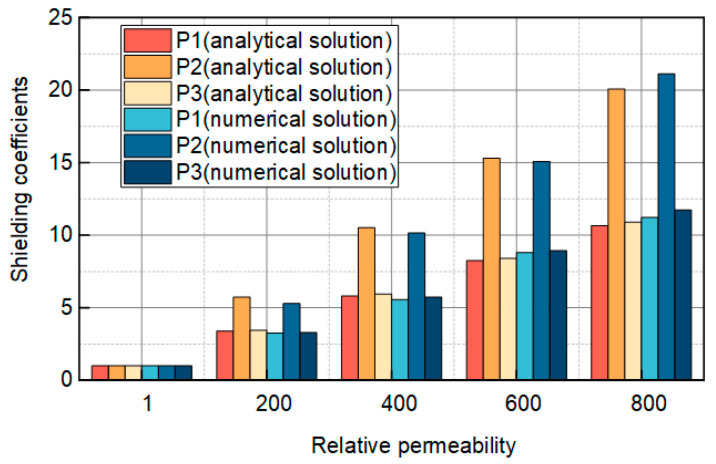
Analytical solution of the shielding coefficients at three points: P_1_, P_2_, and P_3_.

Figure 4 illustrates the analytical and numerical solutions for the magnitude of the shielding coefficient at three locations, P_1_, P_2_, and P_3_, across various relative permeabilities. Each relative permeability is represented by six bars, corresponding to the analytical and numerical solutions for P_1_, P_2_, and P_3_. In the analytical solution for P_1_, the shielding coefficient is 1, 3.39, 5.81, 8.24, and 10.66 when the relative permeability is 1, 200, 400, 600, and 800, respectively. For P_2_, the shielding coefficient is 1.00, 5.73, 10.51, 15.30, and 20.08 when the relative permeability is 1, 200, 400, 600, and 800, respectively. Similarly, for P_3_, the shielding coefficient is 1.00, 3.45, 5.93, 8.41, and 10.89 when the relative permeability is 1, 200, 400, 600, and 800.

In the numerical solution for P_1_, the shielding coefficient is 1, 3.26, 5.56, 8.74, and 11.03 when the relative permeability is 1, 200, 400, 600, and 800, respectively, and for P_2_, the shielding coefficient is 1.00, 5.45, 10.15, 15.10, and 21.12 when the relative permeability is 1, 200, 400, 600, and 800. Similarly, for P_3_, the shielding coefficient is 1.00, 3.30, 5.74, 9.06, and 11.33 when the relative permeability is 1, 200, 400, 600, and 800. From the above data, the shielding coefficient magnitude increases with the relative permeability increase, but the increase’s magnitude becomes smaller. Secondly, in the case of the same relative permeability, the shielding coefficient considering only the outer steel tape is slightly smaller than the shielding coefficient considering only the inner steel tape, and both are much smaller than the shielding coefficient considering the double-layer steel tape.

The magnitude of the error is

(28) $$E=\frac{S_{n}-S_{a}}{S_{a}}\times 100\%$$

where *E* is the magnitude of the error, *S*_n_ is the magnitude of the shielding coefficient obtained from the numerical solution, and *S_a_* is the magnitude of the shielding coefficient obtained from the analytical solution. For P_1_, when the relative permeability is 200, 400, 600, and 800, the magnitude of error between the analytical and numerical solutions is 3.8%, 4.3%, 5.7%, and 3.4%. For P_2_, the magnitude of error between the analytical and numerical solutions is 4.8%, 3.4%, 1.3%, and 5.1% when the relative permeability is 200, 400, 600, and 800. For P_3_, the magnitude of errors between the analytical and numerical solutions are 4.3%, 3.2%, 5.4%, and 3.9% when the relative permeabilities are 200, 400, 600, and 800. The maximum error between the analytical and numerical solutions is around 5%, while the rest are below 5%.

### 2.3. Simulation of Magnetic Fields on Cable Surfaces

The surface structure of a three-core cable is shown in Figure 3. The cable comprises seven layers: copper, an inner semiconductor layer, cross-linked polyethylene insulation, an outer semiconductor layer, a buffer layer, steel tape, and an outer sheath. The dimensions and geometric parameters of each layer are shown in Table 2.

A three-dimensional three-core cable model under the double-layer steel tape-wrapped structure was constructed to conform to the actual case, as shown in Figure 2. The cable is wrapped with double-layer steel tape, and the total length of the model is 100 mm.

(29) $$\left\{\begin{array}{c} \nabla \times H=J+\frac{\partial D}{\partial t}\\ \nabla \cdot B=0 \end{array}\right.$$

In the equations, *H* represents the magnetic field intensity vector, *J* is the conduction current density vector, and *D* is the electric displacement vector. The constitutive equation is given by:

(30) B=μH

where *μ* is the relative permeability, the distribution of magnetic potential on the surface of the three-core cable can be established by the two-bit method under the Lorentz norm constraint.

**Figure 5:**
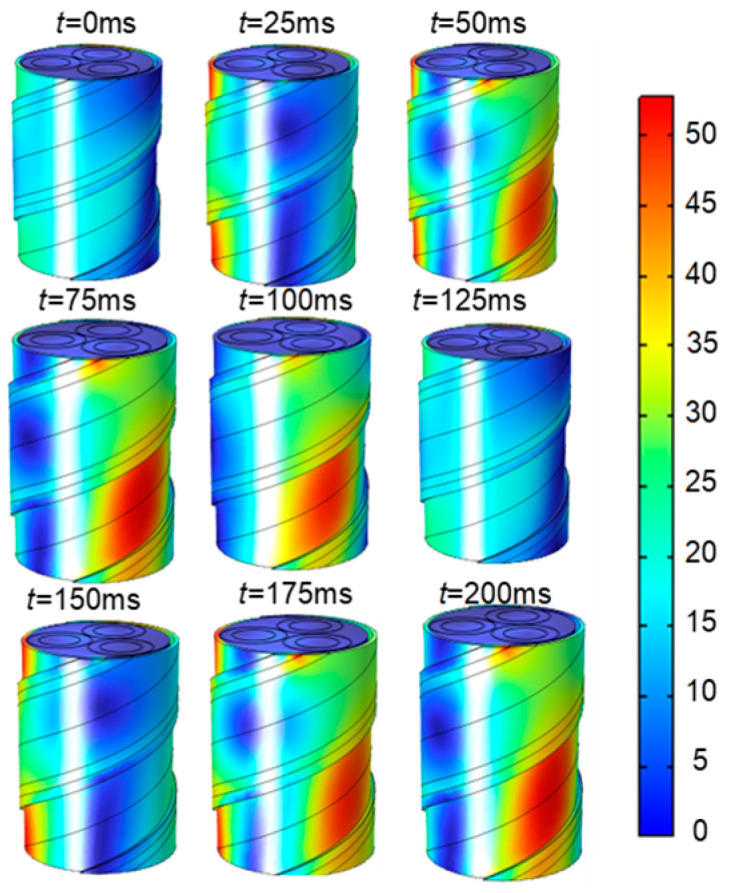
Magnetic induction intensity within a cycle.

The assumption of the quasi-static magnetic field can be established for the three-core cable under industrial frequency current. In the simulation model, a three-core cable is fed with an industrial frequency three-phase alternating current with an RMS value of 200 A. It is assumed that the electric and magnetic vector potentials at infinity are zero and that the outer surface of the cable is electrically and magnetically insulated. The relative permeability of the steel tape is set to 400. The magnetic field distribution in one industrial frequency cycle of a three-core cable is obtained by simulation. As shown in Figure 5, the magnetic induction intensity of the cable surface rotates with the current phase change.

**Figure 6:**
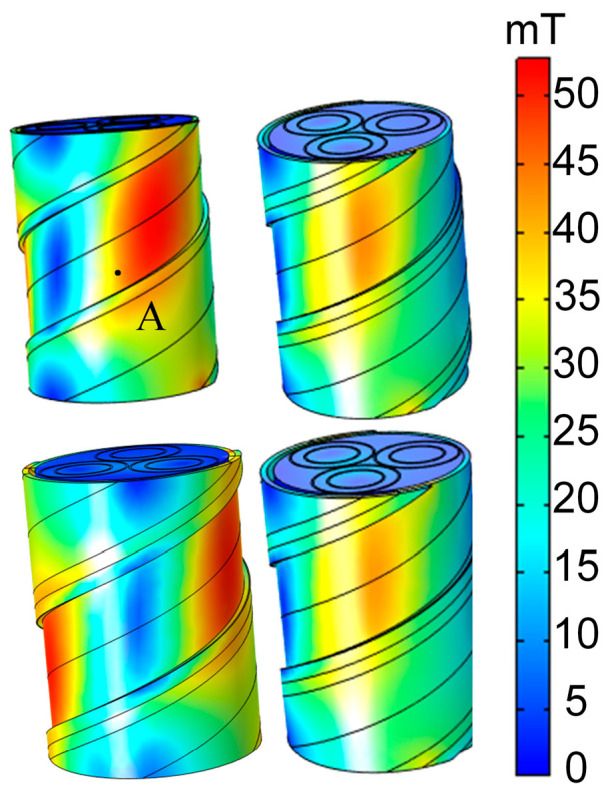
The magnetic induction intensity under different thicknesses of the steel tape shielding layer.

Variations in the structure of three-core cables can influence the magnitude of the magnetic field. This study examines the impact of the double-layer steel tape’s thickness on the cable surface’s magnetic field. Figure 6 depicts the magnetic induction intensity on the cable surface for steel tape thicknesses of 1.5 mm, 1.7 mm, 1.9 mm, and 2.1 mm while keeping the distance of the 6 sensors from the center of the cable constant and their position unchanged.

To compare more intuitively the effect of different steel tape shielding layer thicknesses on the shielding coefficient of the steel tape, any point A on the surface of the steel tape is selected, as shown in Figure 6. When the thickness of the steel tape shielding layer is 1.5 mm, 1.7 mm, 1.9 mm, and 2.1 mm, the magnitude of the magnetic induction intensity *B* at point A is 0.088 mT, 0.085 mT, 0.078 mT, and 0.066 mT, respectively. It can be seen that, with the increase of the thickness of the steel tape shield of the cable, the magnitude of the magnetic induction intensity of the surface of the cable decreases, and the shielding coefficient of the steel tape increases. The tendency to increase the shielding coefficient of the steel tape increases with the thickness of the steel tape increases.

This paper presents a theoretical formula for the shielding coefficient of the steel strip that excludes the eddy current effects of both the conductor and the steel strip. To ascertain the impact of eddy current effects on the shielding coefficient, these effects are incorporated into the simulation.

**Figure 7:**
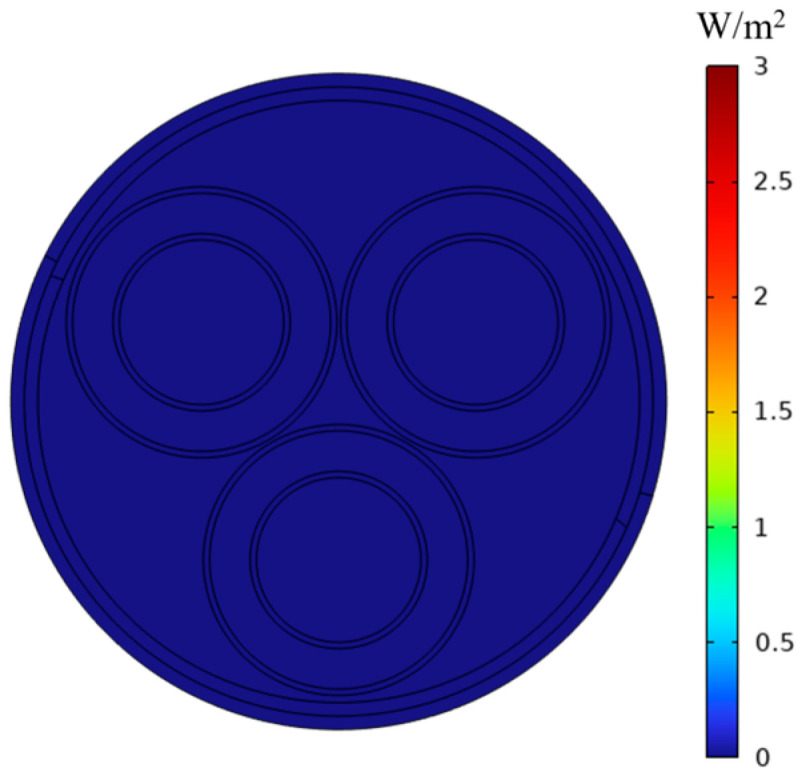
Without considering vortex effects.

**Figure 8:**
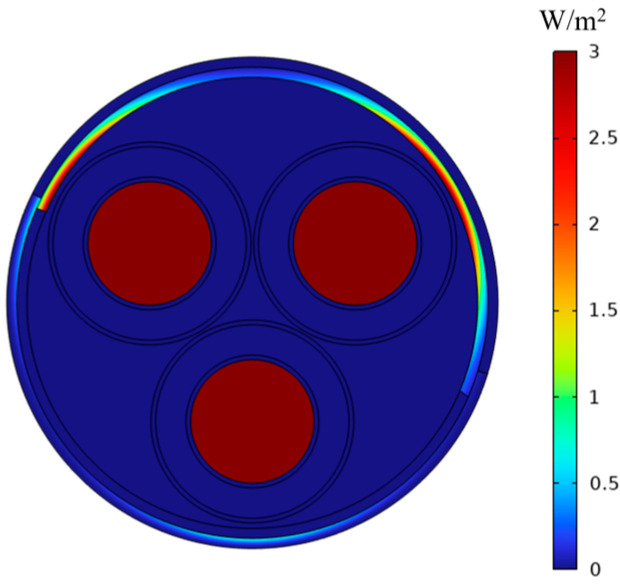
Considering vortex effects.

Figure 7 and Figure 8 illustrate the eddy current loss diagrams on the cable surface, without and including eddy current effects, respectively. In Figure 7, the average eddy current loss of the steel strip is zero, and at this moment, the shielding coefficient at position P1 is 5.81. In Figure 8, the average eddy current loss of the steel strip is 3 W/m^2^, while the shielding coefficient at position P1 is 5.81. Consequently, it can be inferred that the eddy current loss does not influence the magnitude of the shielding coefficient of the steel strip in a low-frequency magnetic field.

## 3. A Numerical Calculation Method for Three-Phase Current Based on the Differential Evolution Algorithm

Obtaining the analytical solution of the nonlinear system of equations is challenging because of the presence of sine and square terms. In the current measurement system, continuous sampling is used to collect multiple measurements of magnetic induction intensity on the surface of three-core cables. These measurements are known quantities that can be used to solve nonlinear equations using numerical methods. The unknown quantity remains constant throughout the measurement process.

This study addresses the issue of finding the numerical solution to a set of nonlinear equations by converting it into an optimization problem. The best solution to the equations is obtained using the differential evolution algorithm through several iterations. Before utilizing the differential evolutionary algorithm, it is necessary to generate the fitness function. This function is constructed using the theoretically computed and measured magnetic field values at six designated measurement points. The resulting fitness function is denoted as *F*:

(31) $$F=\sum_{S=1}^{6}{\left(B_{S}-{B_{S}}^{*}\right)}^{2}$$

*B*_S_ is the theoretically calculated magnetic field value at the measurement point, and *B*_S_^*^ is the measured value of the sensor at the corresponding measurement point. Optimizing the fitness function by determining the optimal three-phase current values, *F* represents the most favorable solution for the current in the system of equations.

The optimization problem fulfills the limitations imposed by the fitness function.

(32) $$\left\{\begin{array}{c} I_{\min}\leq I_{A},I_{B},I_{C}\leq I_{\max}\\ 0\leq \alpha \leq 2\pi \end{array}\right.$$

*I*_A_, *I*_B_, and *I*_C_ are the instantaneous values of the three-phase currents to be sought. *I*_min_ is the lower limit of the current, and *I*_max_ is the upper limit of the current.

The differential evolutionary algorithm is an intelligent optimization search strategy that relies on the cooperation and rivalry among individuals in a population. In contrast to evolutionary computation, it maintains a global search strategy that relies on population, decreases the complexity of evolutionary computation operations, and possesses a distinctive memory capability to dynamically monitor the present search situation with a robust global convergence ability. From a mathematical perspective, it can be described as a stochastic search algorithm. From an engineering perspective, it can be seen as an adaptable iterative optimization process. Furthermore, the differential evolutionary algorithm can engage in collaborative search by utilizing individual local knowledge and group global information to effectively direct the algorithm in its ongoing search process.

**Figure 9:**
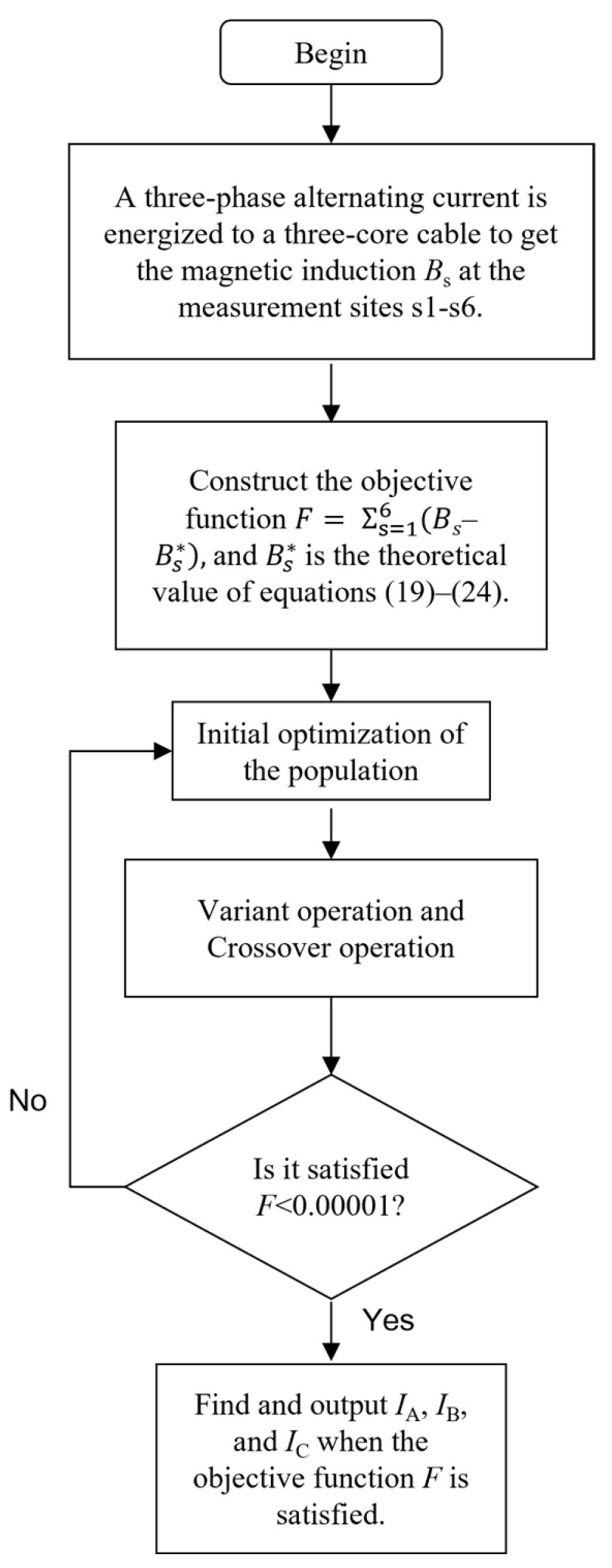
Flow chart of differential evolution algorithm.

The process of the differential evolutionary algorithm is shown in Figure 9.

Typically, the population is initialized by randomly selecting values within the specified boundary restrictions, following a uniform probability distribution:

(33) $$F_{ji,0}=\mathrm{rand}[0,\;1]\cdot (F_{j}^{U}-F_{j}^{L})+F_{j}^{L}$$

where *i* = 1, 2, …, *NP*; *NP* is the population size, *j* = 1, 2, …, *m*; *m* is the dimension of the solution space, which takes the value of 4. rand [0, 1] denotes the random number generated uniformly between [0, 1], *F_ji_*_,0_ is the initialized individual, and *F_j_*^L^ and *F_j_*^U^ are the lower and upper bounds of the parameter variables respectively.

The variation operation produces novel parameter vectors by combining the weighted difference vectors between two individuals from the population and a third individual. During the variation operation, for each target vector *F_i_*_,*G*_ (*i* = 1, 2, …, *NP*), the mutation vector of the differential evolution algorithm is:

(34) $$F_{i,G+1}=F_{r_{1},G}+K\cdot (F_{r_{2},G}-F_{r_{3},G})$$

where *G* is the number of evolutionary iterations, the randomly chosen ordinal numbers *r*_1_, *r*_2_, and *r*_3_ are not the same as each other, and the variation operator *K* ∈ [0, 2] is a real constant factor used to control the scaling of the bias variables.

A crossover operation is implemented to enhance the diversity of parameter vectors. This involves combining the parameter vectors obtained from the variation operation with the predetermined target parameter vectors to create test vectors. The test vectors are then evaluated by comparing their target function values with those of the target parameter vectors. The vector that yields a smaller target function value is selected. In a problem with boundary condition constraints, it is crucial to ensure that the parameter values used to create new individuals fall within the feasible range of the problem. This allows the boundary absorption process to occur, where any values of individuals exceeding the boundary constraints are adjusted to the nearest boundary values. The iteration termination condition depicted in the figure is to reach the predetermined maximum number of iterations and ultimately produce the ideal solution.

This paper provides an example of setting a 10 kV three-core cable when the phase current is in equilibrium. In the differential evolutionary algorithm, the most important parameters are the population size, the maximum number of iterations, and the scaling factor; the more significant the population size and the maximum number of iterations, the better the convergence effect of the objective function, the higher the accuracy of the inversion, but the calculation will be longer. The smaller the scaling factor is, the better the variation effect is, the better the convergence effect is, the higher the inversion accuracy is. The calculation time will be longer at the same time. In the simulation, the population size was set to 100, the maximum number of iterations was set to 1000, and the scaling factor was set to 1. Assume that *i*_a_(*t*), *i*_b_(*t*), and *i*_c_(*t*) are the instantaneous values of A, B, and C phase currents at the moment *t*, the frequency is 50 Hz, the initial phase angle of the A phase is 0°, and there is an angular difference of 120° between the three phases. This work utilizes Equations (19)–(24) to replace the phase current with the magnetic induction intensity at six specific measurement points on the cable surface. The phase current is then determined through inversion using the differential evolution approach. The process of calculating the current by inversion aims to determine the magnitude and phase of the three-phase current. This involves inverting the three-phase current value measured by the magnetic sensor every 1 ms in a cycle and fitting the current values at each moment to obtain the magnitude and phase curves of the three-phase current. The equation utilized in this paper for fitting purposes is:

(35) I=a+bcos(ωt)+csin(ωt)

where *I* is the phase current acquired by inversion, the angular velocity of the IF current, denoted as *w*, is set to 100*π*. Variety a is the fitting DC bias current. The cosine coefficient is denoted by *b*, while the sine coefficient is denoted by *c*. The current’s amplitude (*I_m_*) and phase (*φ*) can be determined by utilizing the values of *b* and *c* in Equation (35), as demonstrated in Equation (36).

(36) $$\left\{\begin{array}{c} I_{m}=\sqrt{b^{2}+c^{2}}\\ \varphi =arctan(\frac{b}{c}) \end{array}\right.$$

The amplitude and phase errors of the phase currents are:

(37) $$\left\{\begin{array}{c} Absolute\;value\;of\;amplitude\;error=\left|I_{m}-I_{0}\right|\\ Absolute\;value\;of\;phase\;error=\left|\varphi -\varphi_{0}\right| \end{array}\right.$$

*I_m_* and *φ* represent the magnitude and phase of the inversion determined in Equation (37), and *I*_0_ and *φ*_0_ represent the magnitude and phase of the initial simulation setup.

**Figure 10:**
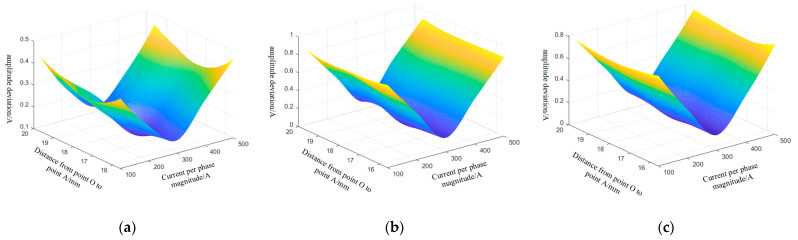
Differential evolution algorithm inverted absolute values of three-phase current magnitude error: (**a**) phase A current magnitude error, (**b**) phase B current magnitude error, and (**c**) phase C current magnitude error.

**Figure 11:**
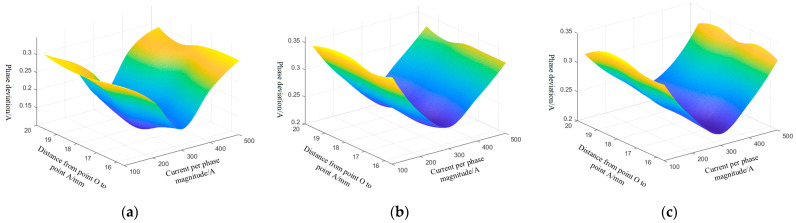
Differential evolution algorithm inverted absolute values of three-phase current phase error: (**a**) phase A current phase error, (**b**) phase B current phase error, and (**c**) phase C current phase error.

In theory, the geometry of a three-core cable should be symmetrical. However, in real operation, external pressures may push out the core wire or cause the cable to deviate from its core, leading to an asymmetrical cable geometry. Consider the extrusion of a biased core into the A-phase core as an example. In this case, when the ra ranges from 15.61 to 19.61 mm, and the three-phase current RMS values range from 100 to 500 A, the amplitude error and phase error of the A, B, and C-phase currents obtained from the inversion of the differential evolution algorithm are shown in Figure 10 and Figure 11.

The differential evolutionary algorithm inversion produces varying amplitude and phase errors, which depend on the RMS current per phase. The highest inaccuracy in the amplitude of the phase A current is 0.46 A, and the maximum error in the phase of the phase A current is 0.32°. The highest inaccuracy in the amplitude of the phase B current is 0.92 A, and the maximum error in the phase of the phase B current is 0.36°. The highest inaccuracy in the amplitude of the phase C current is 0.92 A, and the maximum error in the phase of the phase C current is 0.36°.

## 4. Experiment Platform and Experimental Validation

**Figure 12:**
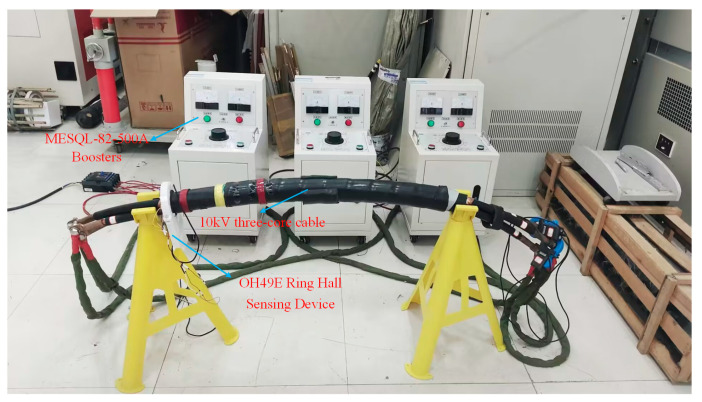
The experimental setup for measuring the phase currents of three-core cables under a double-layer steel tape-wrapped structure.

The experiment platform is shown in Figure 12.

**Figure 13:**
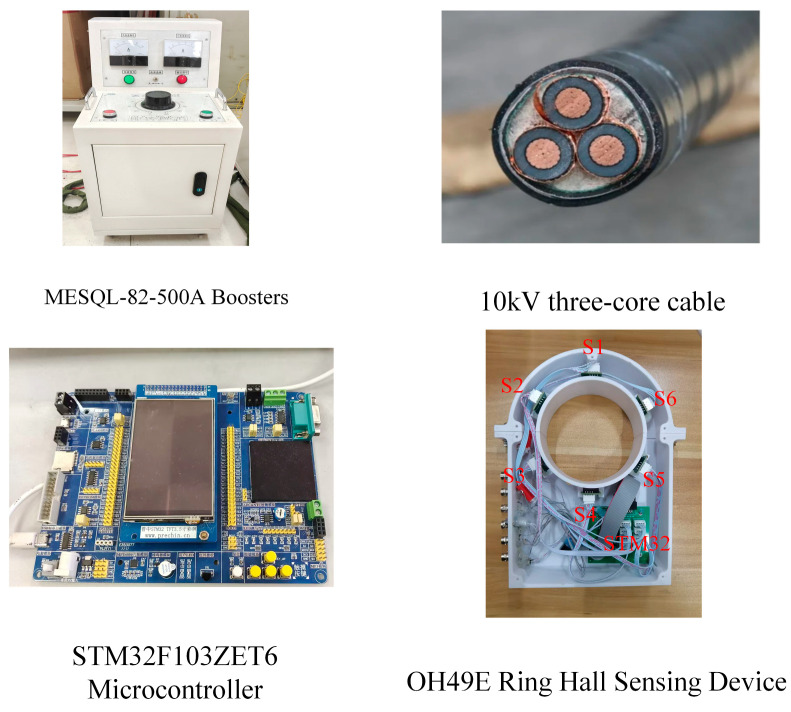
Experimental equipment.

The experiment platform comprises a 10 kV medium voltage three-core cable and experiment equipment. The experiment equipment consists of three single-phase current transformers (MESQL-82-500A), a microcontroller based on the STM32F103ZET6 microcontroller, an annular Hall sensor consisting of six OH49E Hall sensors, and a portable laptop computer (HP-R9000P). Figure 13 illustrates the unique experimental apparatus. All of the above equipment are sourced from Wuhan, China.

The Hall sensing device, depicted in Figure 13, comprises 6 Hall sensors arranged at 60° intervals and an STM32 microprocessor. The hall sensors and microcontroller have been acquired from a vendor. Each sensor is linked to the STM32 microcontroller using a Dupont cable, featuring interfaces, such as VCC (5V power input), GND (ground), AO (analog output), and DO (digital output). Given that magnetic field measurements necessitate real-time acquisition of continuously varying analog signals, we opted to connect the sensor’s AO interface to the STM32’s analog input pins, whereas the DO interface remains unused in this context.

During the experiment, an industrial frequency three-phase current with an RMS value of 200 A was applied to the three-core cable through the transformer. The mutual inductance between the cables results in a change in the output current of the other two phase boosters when the current of one phase booster is adjusted. Consequently, whenever an adjustment is implemented, the currents of the other two phase boosters must initially be maintained constant to ensure a balanced final three-phase output current. Hall sensors are used to sense magnetic fields generated by electrical currents. The experiments were performed during the night. During daylight hours, numerous individuals utilise electricity, resulting in significant voltage fluctuations. During nighttime, electricity consumption diminishes, resulting in reasonably stable voltage fluctuations. A dehumidifier was installed in the laboratory to maintain a relatively dry environment. All experiments were performed at a consistent room temperature wherever feasible. However, replicating these ideal conditions in practical engineering is often difficult; therefore, the following anti-interference strategies are recommended: implementing real-time filtering algorithms in the sensing equipment or encasing it with a compatible steel tape shield. Both methods can significantly improve measurement accuracy in complex situations.

The experiment steps are as follows: First, to measure the magnetic field on the cable surface without a steel tape, part of the length of the cable steel tape on the cable is peeled off, as depicted in Figure 12. Then, a ring Hall sensing device is installed on the surface of the cable, and the six Hall sensors correspond to the six points S1–S6. Then, a three-phase AC is applied to the three-core cable through a current transformer, and finally, the ring Hall sensing device is used to measure the magnetic induction intensity at S1–S6.

**Figure 14:**
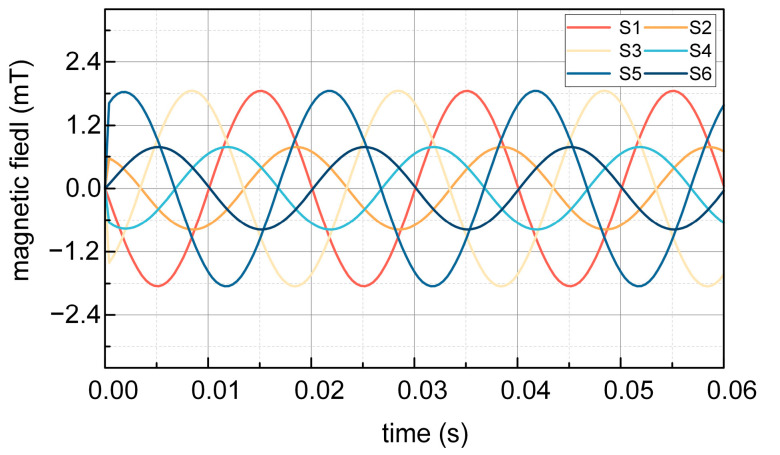
Experiment results of magnetic induction intensity at S1–S6 without steel shielding during the night.

**Figure 15:**
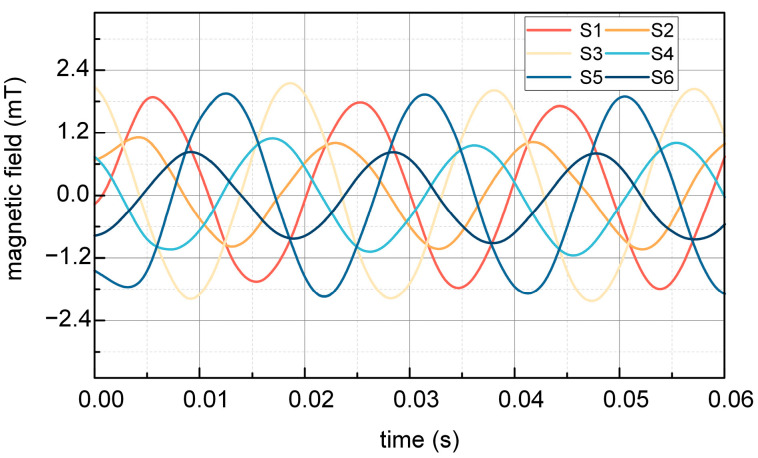
Experiment results of magnetic induction intensity at S1–S6 without steel shielding during the day.

Figure 14 and Figure 15 illustrate the comparison of test results obtained at night and during the daytime, respectively. Waveform analysis reveals two notable distinctions: firstly, the quality of waveforms during daylight is inferior to that observed at night; secondly, the amplitudes of magnetic induction strengths for S1, S3, S5 and S2, S4, S6 in the daytime do not attain the theoretically anticipated levels, in contrast to the nighttime results, which align with theoretical expectations.

The experiment result of magnetic induction intensity at S1–S6 without steel shielding during the night is depicted in Figure 14. It can be seen that the magnetic induction intensity of S1, S3, and S5 measured in the experiment is 1.64 mT, and the magnetic induction intensity of S2, S4, and S6 measured in the experiment is 0.8 mT. From the above results, it can be seen that without the shielding effect of the steel tape, the magnitude of the magnetic induction intensity at S1, S3, and S5 is the same, and the magnitude of the magnetic induction intensity at S2, S4, and S6 is the same.

**Figure 16:**
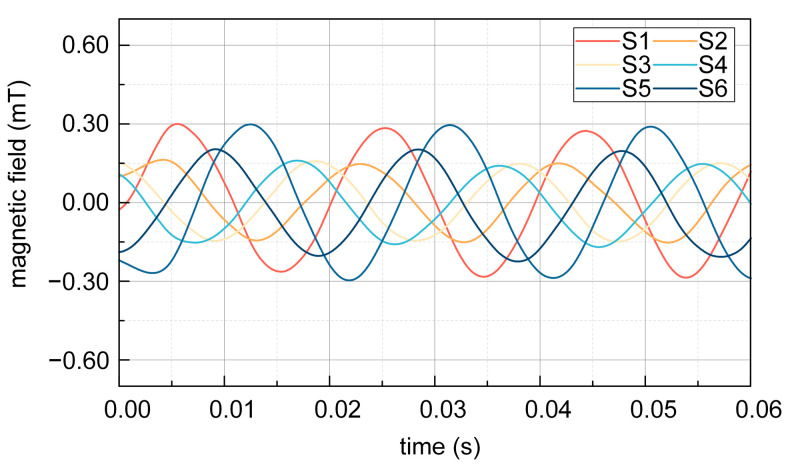
Experiment results of magnetic induction B at S1–S6 with steel shielding during the night.

The experimental result of magnetic induction at S1–S6 with steel shielding during the night is depicted in Figure 16. It can be seen that when affected by the shielding effect of the steel tape, the magnetic induction measured in the experiment is 0.300 mT at S1 and S5, 0.158 mT at S3, 0.160 mT and 0.200 mT at S2 and S6, and 0.156 mT at S4. The magnetic induction intensity at S1, S3, and S5 is not equal, and the magnetic induction intensity at S2, S4, and S6 is not equal under the shielding of the double-layer steel tape, which is caused by the shielding effect of the steel tape at different positions of the sensor.

Once the magnetic induction intensity of the measurement spots impacted by the steel tape’s shielding is determined, the three-phase currents are generated by applying the differential evolution method for inversion. Subsequently, the formula for the inversion current error is defined.

When the maximum number of iterations is 2000, so that the population size is 100, 150, or 200, the absolute value error of the current amplitude and the absolute value error of the phase obtained by the inversion of the differential evolutionary algorithm for phases A, B, and C are shown in Table 3 below.

In the parameter configuration, an increase in population size results in a reduction of amplitude and phase errors derived from the inversion. With a population size of 200, the amplitude error can be reduced to 4.38%, and the phase error can be minimized to 8.01°.

When the population size is 200, so that the maximum number of iterations is 1000, 2000, or 3000, the absolute value error of the current amplitude and the absolute value error of the phase obtained by the inversion of the differential evolutionary algorithm for phases A, B, and C are shown in Table 4 below.

When the maximum number of iterations is increased, the amplitude and phase errors obtained from the inversion are reduced. However, when the maximum number of iterations exceeds 2000, the error reduction is not significant.

In order to highlight the advantages of the differential evolution algorithm, the current amplitude and phase errors obtained from the inversion of the differential evolution algorithm are compared with the particle swarm algorithm and the genetic algorithm.

The amplitude and phase errors obtained from the inversion of these three algorithms are shown in Table 5 below.

Table 5 shows that the amplitude and phase errors obtained from the differential evolutionary algorithm’s inversion are minimized. The amplitude error can be reduced to 4.38%, and the phase error can be minimized to 8.01°. The current amplitude magnitudes derived by inversion for phases A, B, and C are 354.62 A, 354.33 A, and 323.46 A, respectively. The present angles of Phases A, B, and C are −8.21°, −128.01°, and 111.87°. The differential evolution algorithm surpasses particle swarm and genetic algorithms, yielding more minor errors in current magnitude and phase angle. According to Equation (7), it can be seen that the errors mainly come from the following: (1) The relative permeability of the steel tape, contingent upon its kind, is 400; nevertheless, real measurements may exhibit slight deviations from this value. (2) Imprecise measurement of the steel strip thickness in real-world scenarios.

## 5. Conclusions

This article first derives the shielding coefficient formula of the three-core cable shielding layer under a double-layer steel tape-wrapped structure. Then, the shielding coefficient obtained from the numerical and analytical solutions is compared under different relative magnetic permeabilities of the shielding layer. Then, the expression of the magnetic induction intensity *B* at the measurement points on the surface of the three-core cable without shielding was modified to obtain the magnetic induction intensity *B* at the measurement points after shielding with a double-layer steel tape-wrapped structure. Then, a three-core cable model under a double-layer steel tape-wrapped structure was constructed in finite element simulation software. By simulating the surface magnetic field distribution of the three-core cable under a double-layer steel tape-wrapped structure with the condition of three-phase current balance and simulating the impact of different sizes on the shielding coefficient. Ultimately, an experimental platform was built to test the magnetic induction intensity of the measurement spots while being shielded by the steel tape. The three-phase currents were derived by inverting the measured magnetic induction intensity. Conclusion as below:

(1) The error between the shielding coefficient obtained from the numerical solution and the analytical solution under different relative magnetic permeabilities of the shielding layer does not exceed 5%.
(2) The shielding coefficient of the steel tape increases when the relative permeability of the steel tape increases and when the thickness of the steel tape increases. However, as the relative permeability of the steel tape increases, the trend of increasing the shielding coefficient of the steel tape slows down. As the thickness of the steel tape increases, the trend of increasing the shielding coefficient of the steel tape accelerates.
(3) The differential evolution algorithm can accurately invert the three-phase currents. The magnitude of the current amplitude of phase A, phase B, and phase C measured by the differential evolutionary algorithm is 354.62 A, 354.33 A, and 323.46 A, and the magnitude of the current phase is −8.21°, −128.01°, and 111.87°. The amplitude error of the three-phase current is limited to a maximum of 4.83%, while the phase error is limited to a maximum of 8.01°.

## Figures and Tables

**Table 1:** The magnetic induction intensity *B* at P_1_, P_2_, and P_3_.

	*u* = 1	*u* = 200	*u* = 400	*u* = 600	*u* = 800
*B*_P1_	0.528 mT	0.162 mT	0.095 mT	0.060 mT	0.047 mT
*B*_P2_	0.528 mT	0.202 mT	0.052 mT	0.035 mT	0.025 mT
*B*_P3_	0.528 mT	0.160 mT	0.092 mT	0.057 mT	0.045 mT

**Table 2:** The dimensions of each structural component in the three-core cable.

Structure	Radius	Materials
copper	0.528 mm	copper
inner semiconducting layer	9.85 mm	semiconductor
insulation layer	14.35 mm	XLPE
outer semiconducting layer	15.05 mm	semiconductor
copper shielding layer	15.25 mm	copper
steel shielding layer	35.46 mm	steel
outer sheath	36.96 mm	PVC

**Table 3:** Magnitude and phase errors at different population sizes.

Population Size	Error	A	B	C
100	amplitude error phase error	25.35 A 12.56°	23.01 A 12.37°	24.38 A 14.42°
150	amplitude error phase error	17.73 A 10.25°	16.21 A 10.31°	17.33 A 10.21°
200	amplitude error phase error	15.21 A 8.12°	15.33 A 8.01°	15.54 A 8.13°

**Table 4:** Amplitude and phase errors at different maximum numbers of iteration.

Number of Iterations	Error	A	B	C
1000	amplitude error phase error	19.24 A 8.21°	18.52 A 8.32°	19.37 A 8.21°
2000	amplitude error phase error	15.21 A 8.12°	15.33 A 8.01°	15.54 A 8.13°
3000	amplitude error phase error	15.09 A 8.25°	15.44 A 8.05°	16.37 A 8.20°

**Table 5:** Amplitude and phase errors at different algorithms.

Algorithm	Error	A	B	C
differential evolution algorithm	amplitude error phase error	15.21 A 8.12°	15.33 A 8.01°	15.54 A 8.13°
particle swarm algorithm	amplitude error phase error	18.32 A 10.21°	18.41 A 10.45°	17.90 A 11.35°
genetic algorithm	amplitude error phase error	21.45 A 12.13°	20.14 A 11.58°	21.97 A 12.75°

## Data Availability

Data are contained within the article.
